# T Cell Migration in Rheumatoid Arthritis

**DOI:** 10.3389/fimmu.2015.00384

**Published:** 2015-07-27

**Authors:** Mario Mellado, Laura Martínez-Muñoz, Graciela Cascio, Pilar Lucas, José L. Pablos, José Miguel Rodríguez-Frade

**Affiliations:** ^1^Department of Immunology and Oncology, Centro Nacional de Biotecnología, Consejo Superior de Investigaciones, Madrid, Spain; ^2^Grupo de Enfermedades Inflamatorias y Autoinmunes, Instituto de Investigación Sanitaria Hospital, Madrid, Spain

**Keywords:** chemokines, cytokines, rheumatoid arthritis, inflammation, cell migration

## Abstract

Rheumatoid arthritis (RA) is an autoimmune disease characterized by chronic inflammation in joints, associated with synovial hyperplasia and with bone and cartilage destruction. Although the primacy of T cell-related events early in the disease continues to be debated, there is strong evidence that autoantigen recognition by specific T cells is crucial to the pathophysiology of rheumatoid synovitis. In addition, T cells are key components of the immune cell infiltrate detected in the joints of RA patients. Initial analysis of the cytokines released into the synovial membrane showed an imbalance, with a predominance of proinflammatory mediators, indicating a deleterious effect of Th1 T cells. There is nonetheless evidence that Th17 cells also play an important role in RA. T cells migrate from the bloodstream to the synovial tissue via their interactions with the endothelial cells that line synovial postcapillary venules. At this stage, selectins, integrins, and chemokines have a central role in blood cell invasion of synovial tissue, and therefore in the intensity of the inflammatory response. In this review, we will focus on the mechanisms involved in T cell attraction to the joint, the proteins involved in their extravasation from blood vessels, and the signaling pathways activated. Knowledge of these processes will lead to a better understanding of the mechanism by which the systemic immune response causes local joint disorders and will help to provide a molecular basis for therapeutic strategies.

## Rheumatoid Arthritis

Incorrect resolution of inflammation underlies pathologies of clinical importance, including cancer, atherosclerosis, and rheumatic diseases, and precise understanding of inflammatory responses is a major challenge to medical science. Of these conditions, rheumatoid arthritis (RA) is an enormous economic and social problem, highly prevalent in industrialized countries (0.5–1%, with two- to threefold greater incidence in women), resulting in disability, loss in quality of life, and reduced life expectancy.

Rheumatoid arthritis is a systemic autoimmune disease, characterized by non-organ-specific autoantibody production and chronic inflammation of synovial tissues, leading to cartilage and bone destruction. During disease development, other organs can also become inflamed, and as a consequence, systemic cardiovascular, pulmonary, and skeletal complications frequently appear (1). Little is known of the initiating events or factors that perpetuate RA, but advances in understanding the pathogenesis of the disease have contributed notably to development of new therapies. RA is a polygenic disease that involves complex interactions between genetic and environmental factors. The long-established association of RA patients with the human leukocyte antigen (HLA)-DRB1 locus suggests the influence of T cell selection and antigen presentation in the induction of autoreactive immune responses (2–4). Other genetic risk alleles/factors in RA include immune regulation (CD28), NF-κB-dependent signaling (TRAF1), control of T cell activation (PTPN22), and functional differentiation (CTLA-4) (5–9) (Table 1). Many cytokines, chemokines, growth factors, intracellular signaling molecules, and transcription factors have also been implicated in RA pathogenesis (10, 11).

**Table 1:** **Most relevant genes with single-nucleotide polymorphisms associated with susceptibility to rheumatoid arthritis and their functional role**.

Gene	Location	Function
*HLA-DRB1*	6p21.3	Encodes the cell surface complex for antigen presentation
*PTPN22*	1p13.2	Encodes a tyrosine phosphatase involved in the immune response
*STAT4*	3q32.2	Encodes a transcription factor implicated in cytokine and chemokine signaling
*TRAF1*	3q33.1	Encodes a regulator of the TNFα receptor
*PAD14*	1p36.13	Encodes a peptidylarginine deiminase that catalyzes conversion of arginine to citrullin
*IRF5*	7q32.14	Encodes a member of the interferon regulatory factor
*FcGR2a*	1q23.2	Encodes the low affinity IgG Fc receptor
*IL2RA*	10p15.1	Encodes the high affinity IL2 receptor
*CD40*	20q13.2	Encodes a costimulatory molecule that enhances B/T cell interactions
*CD28*	2q33.2	Encodes a negative regulator of DC/T cell interaction
*CCL21*	3q13.3	Encodes a chemokine implicated in lymphocyte homing
*CCR6*	6q27	Encodes a chemokine receptor implicated in Th17 recruitment

**Figure 1:**
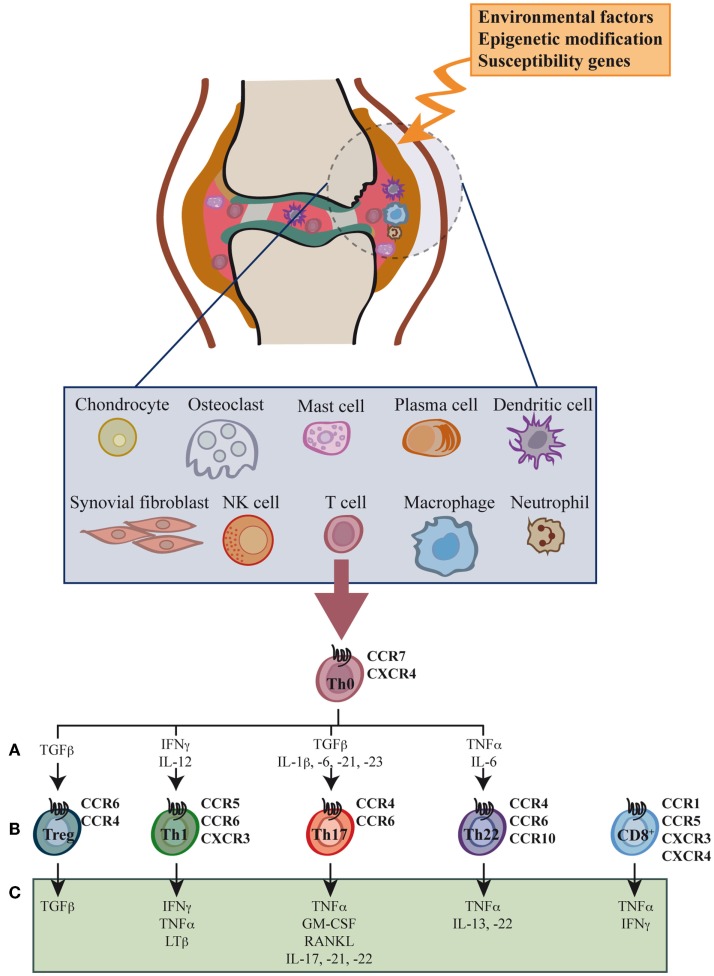
**Cell types, cytokines, and chemokine receptors involved in rheumatoid arthritis development**. Environmental factors and susceptibility gene interactions promote loss of tolerance to citrullinated self proteins generated by post-translational modifications. Co-stimulation-dependent interactions among DCs, T cells, and B cells generate an autoimmune response to these self proteins. This inflammatory process occurs primarily in the lymph node, but also in the inflamed joint. Adaptive and innate immune cells are attracted to the joint where immune pathways integrate to promote tissue remodeling and damage. Positive feedback loops mediated by interactions among leukocytes, synovial fibroblasts, chondrocytes, and osteoclasts, together with the molecular products of damage, drive the chronic phase in rheumatoid arthritis (RA) pathogenesis. High levels of activated memory CD4^+^ and CD8^+^ T cells differentiated through cytokine stimulation of naïve cells infiltrate the synovia **(A)**. RA was classically considered a type 1 T helper (Th1)-mediated disease, but today data indicate that type 17 T helper cells (Th17) are more important in its promotion. Evidence shows that type 22 T helper cells (Th22) also contribute to RA pathogenesis. Function of regulatory T cells (Treg) is also reduced and effector cell resistance to suppression thus helps to alter the immune balance in inflamed joints. The figure shows the chemokine receptor expression pattern **(B)** and the main secreted cytokines **(C)** associated with each T cell subtype.

Synovial inflammation, or synovitis, results from leukocyte infiltration of the synovial compartment, enabled by increased expression of adhesion molecules and chemokines in the endothelium (Figure 1) (12–14). The cellular infiltrate includes granulocytes, monocytes/macrophages, NK cells, B cells, and especially CD4^+^ and CD8^+^ T cells, all leading to the production of large amounts of chemokines and proinflammatory cytokines (15–20).

The role of these infiltrating cells is poorly understood. CD4^+^ T cells, but not CD8^+^ T cells or B cells, are necessary for disease initiation (21), but not for the inflammatory phase of the disease; hyperactivation of the immune response and the presence of autoantibodies in the synovial microenvironment are sufficient for disease development (22, 23). CD4^+^ T cell depletion using specific antibodies suppresses autoantibody production and reduces disease severity in the collagen- or antigen-induced arthritis models in rodents (CIA and AIA, respectively) (22, 24–27). Disease can nonetheless be induced in murine models of collagen antibody-induced arthritis (CAIA) in the absence of T cells (28). These differences indicate that murine RA models reflect only partial steps in the human disease and underline the limitations of the *in vivo* models (29). The limited effectiveness of T cell-depleting strategies (22) in clinical studies is probably due to immunogenicity and poor reconstitution of the immune system and emphasizes the restraints of *in vitro* testing and *in vivo* models (29). In contrast, therapies that block T cell co-stimulation are very effective at both early and advanced disease stages (30, 31).

Although RA was generally considered dependent on IFN-γ-producing Th1 cells, recent evidence indicates an important role for Th17 cells in RA development (11, 22) (Figure 1). Cytokines expressed by these cells (IL-17, GM-CSF, IL-22) are associated with synovial inflammation, mainly through their effect on neutrophil activation (10, 32). IFN-γ levels are not high in synovial membranes of RA patients, and RA development is reported in IFNγ-deficient mice (33, 34). In contrast, IL-17 deficiency mitigates arthritis development, as seen in mice that lack IL-17A (35, 36) or those treated with anti-IL-17-blocking antibodies (36, 37); IL-17 overexpression exacerbates disease progression and induces a chronic, erosive form of the disease (38). Although not the site of naïve T cell priming, CD4^+^ T cell commitment might occur at the inflamed joints that also have larger numbers of activated macrophages and dendritic cells (DCs) (15, 20). In mice, Th17 cell commitment requires IL-6, TGF-β, and IL-23 expression. In human beings, Th17 polarization depends on IL-1β, IL-6, IL-21, and IL-23, but the role of TGF-β is unclear (39). All of these cytokines are produced by tissue-resident macrophages, although the importance of specific DC subsets in T cell priming and polarization is becoming evident. An increase in Th17 cells is induced by monocyte-derived DC and CD1c^+^ myeloid DC, both found at high frequency in RA patient synovial fluid (40, 41), and by human inflammatory DC (42). DC from RA patient synovial fluid secrete higher levels of CCL17 than DC in peripheral blood; this chemokine could contribute to recruitment of CCR6^+^ cells, including Th17, to the inflamed joint (41). In mice, disruption of immune homeostasis by mucosal DC depends on the presence of commensal bacteria (43). Triggering of Toll-like receptors by intestinal flora contribute to RA progression by altering the Th17/regulatory T cells (Treg) balance, suggesting a role for the microbiota in Th17 response induction in RA (44, 45).

IL-17 has pleiotropic effects on many cell types, induces migration of innate immune cells, increases production of cytokines, chemokines, and matrix metalloproteases (46, 47), and enhances germinal center formation in animal models (48, 49), all of which contribute to the initiation and inflammatory phases of RA. In addition, IL-17 drives osteoclastogenesis, leading to bone resorption (50). Despite success in other types of arthritic diseases, IL-17-blocking strategies have thus far been less effective than anticipated in RA; this raises questions regarding the contribution of Th17 cells to RA development in human beings (51).

Activated naïve CD4^+^ T cells differentiate to IL-22-producing Th22 cells in the presence of IL-6 and TNFα. Similar to Th17, Th22 cells express CCR4 and CCR6, as well as CCR10 (52). Th22 cells are implicated in epidermal immunity, although their role in RA is unclear (Figure 1). Th22 cell frequencies are increased in peripheral blood from RA patients (53), and their percentages correlated positively with plasma IL-22 levels in these individuals (54). These observations coincide with reports that link IL-22 with RA activity and bone damage (55, 56). Results in animal models are also debated, whereas IL-22^−/−^ mice show reduced incidence of CIA (57), recombinant IL-22 administration prior to arthritis onset reduces disease severity (58), suggesting a dual role for IL-22 during RA development (59).

The inflammatory environment also induces Treg expansion, and large numbers of proliferating Treg cells can be detected in the inflamed joints of patients (19). Data from murine models indicate that TNFα can boost Treg cell expansion (60), and thus protect mice from induction of autoimmune diseases. In man, however, TNFα has a negative effect on Treg cell expansion *in vitro* and *in vivo* (61, 62). The data indicate that Treg, Th1, and Th17 cells are the key T cell subsets in joint inflammation (Figure 1).

T cell plasticity is described in many inflammatory conditions. IL-1β and IL-6 downregulate FoxP3 expression, thus reducing Treg suppressive function (63, 64). In the inflamed synovium, TNFα promotes FoxP3 dephosphorylation and impaired Treg function (65). In these conditions, Treg cell dysfunction correlates with increased IL-17^+^ and IFN-γ^+^ CD4^+^ T cell numbers (65). In these inflammatory conditions, Treg cells differentiate into IL-17- and IFN-γ-producing effector cells (66, 67). Th17 cells in joints also show plasticity, as they co-express IFN-γ and transcription factors characteristic of Th17 (RORC) or Th1 (T-bet) cells; when cultured in synovial fluid, Th17 cells are reported to convert to Th1 cells (68–70). It is thus possible that Th17 cells give rise to a population of Th1 cells in inflamed joints, which could explain the pronounced Th1 responses in the inflamed synovium (68, 71, 72).

With activated macrophages and granulocytes, these T cell subsets contribute to the production of the proinflammatory cytokine cocktail that aggravates RA. TNFα and its receptors are expressed in human rheumatoid joint tissue (73, 74) and have a key role in RA, as they participate in cartilage (75) and bone degradation (76), and also promote IL-1, IL-6, and IL-8 production (77). In the CIA model of RA, administration of a specific anti-TNFα monoclonal antibody (mAb) after disease onset ameliorated inflammation and joint damage (27, 78). This therapy restores Treg cell function in RA patients (65), and antibody-based therapies that target TNFα are widely and successfully used in the clinic (79). IL-1α and IL-1β are also expressed in abundance in the synovial membrane (80), and IL-1Rα-deficient mice develop spontaneous arthritis, mediated in part by amplification of Th17-dependent inflammation (81). IL-18, another member of the IL-1 superfamily, is detected in RA synovium (82); symptoms are reduced for CIA in IL-18-deficient mice, as are those of RA in rodent models when IL-18 is blocked using neutralizing antibodies (83, 84). Given its pleiotropic functions, IL-6 is also important in RA; it regulates the maturation and activation of B and T cells, macrophages, osteoclasts, chondrocytes, and endothelial cells and has broad effects on hematopoiesis in the bone marrow. IL-6 deletion protects DBA/1 mice from CIA, and neutralization of IL-6 reduces the disease (85, 86). IL-12 is the main stimulator of IFN-γ production and of development of Th1 autoimmune responses (87); although the use of neutralizing antibodies in a murine CIA model attenuates symptoms, prolonged treatment worsens the disease (88).

## T Cell Trafficking to the Synovium

**Figure 2:**
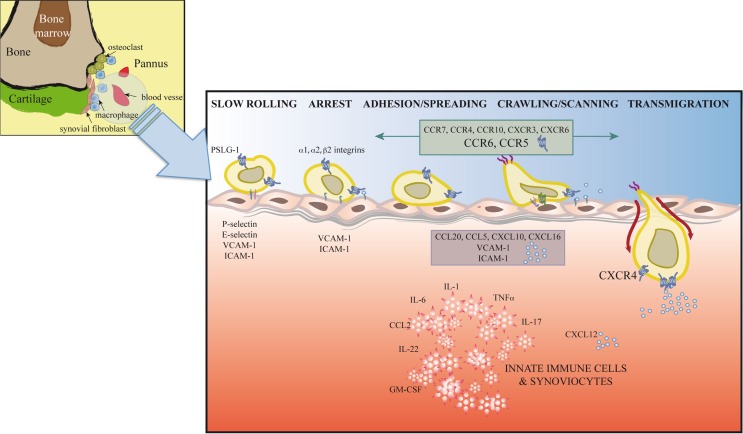
**Extravasation model for T cells at the inflamed joint**. In response to proinflammatory mediators, leukocytes and vascular cells are activated. Among other immune cells, T cells (Th1, Th17, Treg, and possibly Th22) initiate a serial cascade (rolling, arrest, spreading, crawling, and transmigration) and eventually extravasate from blood vessels to the inflamed joint. The figure shows inflammatory cytokines, selectins, integrins, adhesion molecules, chemokines, and chemokine receptors involved in T cell recruitment to and retention in the joint.

Synovial inflammation in RA is partially dependent on migration of inflammatory cells, their retention at the inflammation site, and insufficient apoptosis of chronic inflammatory and stromal cells (89). T cell trafficking to the sites of inflammation is enabled by local activation in synovial vessels of the mechanisms necessary for leukocyte recruitment; alterations in these mechanisms can lead to chronic inflammation and autoimmunity (Figure 2).

The leukocyte adhesion cascade is a multistep process that requires the coordinated action of rolling, adhesion, and transmigration events. This cascade is currently seen as the result of a chain of events initiated by leukocyte rolling along the endothelium, followed by their activation and adhesion to endothelial cells, and finally, migration to the target tissue (13, 14). Only those leukocyte subsets that express the appropriate set of adhesion molecules and chemoattractants will be recruited to specific sites. Leukocyte rolling is mediated by selectins, which are expressed by most leukocyte populations (L-selectin) and by inflamed endothelial cells (E- and P-selectins) (90) (Figure 2). Rolling involves selectins and P-selectin glycoprotein ligand-1 (PSGL-1), expressed by leukocytes and inflamed endothelial cells, as well as other glycosylated ligands (91). Interaction between PSLG1 and L-selectin is needed for leukocyte–leukocyte interactions that enable leukocyte tethering and adhesion to the inflamed endothelium in conditions of blood flow (91). E-selectin, which is upregulated in the inflamed synovium, is decreased after TNF-α therapy (92). Although serum levels of P- and L-selectin are reported to be increased in RA patients (93), the use of blocking antibodies and selectin-deficient mice only correlate P-selectin levels with disease activity (94, 95).

Integrins also participate in rolling and are responsible for firm leukocyte adhesion and arrest (13, 14) (Figure 2). Unlike circulating leukocytes, in the synovia, these populations express high levels of specific subsets of activated integrins. These integrins interact on the endothelial cell surface with ICAM1 or VCAM1, adhesion molecules that belong to the immunoglobulin superfamily, a prerequisite for cell extravasation. Distinct cell types express specific integrins. Whereas α1 are strongly expressed in activated CD4^+^ and CD8^+^ T cells, Th17 cells upregulate α2 integrins, a costimulatory molecule thought to be necessary for IL-17 production (96, 97). This specific integrin upregulation is fostered by proinflammatory cytokines in the synovia such as IL-1 or TNFα (98, 99) and determines cell localization in the inflamed joint. In addition to its effect on cell adhesion, interaction between integrins and their ligands, including fibronectin, collagen, VCAM-1, or degraded cartilage, also induces cell proliferation, cytokine production, and angiogenesis, contributing to disease development (96, 100, 101). Antagonists of integrins and their ligands thus prevent inflammation and angiogenesis in the murine CIA model (97, 102).

Stimulation by IL-1, TNFα, or IFNγ induces high levels of soluble and endothelium-bound ICAM-1, the β2 integrin ligand, in RA patient synovia (98, 103) (Figure 2). The role of ICAM-1 in RA is supported by lower disease activity in a CIA model in ICAM-1-deficient mice and by clinical studies that showed beneficial effects of a blocking anti-ICAM-1 mAb in early RA (104, 105).

Endothelial cells respond to inflammatory conditions by promoting expression of adhesion molecules and chemoattractants that bind directly or indirectly to glycosaminoglycans (GAGs) on the endothelial cell membrane. Integrin activation, initiated through chemoattractant-mediated inside–out signaling, induces the conformational changes responsible for the increased ligand-binding affinity needed for leukocyte arrest. The chemokine-activated signaling pathways responsible for integrin regulation and activation are not yet completely understood. G protein-dependent signaling through small GTPases is involved in rapid LFA-1 activation (106, 107). Recent reports suggest the existence of G protein-independent mechanisms that link Janus kinase (JAK)-mediated chemokine signaling with integrin activation via RhoA, RAC1, and Rap1 (108–110).

Transendothelial migration following chemotactic gradients is the final step in leukocyte migration through paracellular or transcellular pathways into inflamed tissues (Figure 2). Studies using knockout mice indicate a role in leukocyte transmigration of endothelial cell junction molecules such as PECAM1, ICAM2, JAMA, and ESAM (13).

Specificity of this process is achieved through carefully regulated cell signatures, that is, differential expression of the distinct components of this leukocyte adhesion cascade, including selectins, integrins, chemokines, and their respective ligands or receptors. For example, naïve T cells express low LFA-1, α4 integrin, and CCR7 levels, which allow cell recirculation through lymphoid tissues but it is insufficient to permit cells entry into inflamed tissues. In contrast, T effector and memory cells with elevated expression of LFA-1, α-integrins, E- and P-selectin ligands, CCR1, CCR5, and CXCR3 enter these tissues. The role of chemokines in T cell recruitment to the synovia is analyzed in detail in the next section.

These data indicate the potential of T cell migration inhibitors as targets for anti-inflammatory therapy. Whereas the limited number of selectins and integrins raises possible specificity problems of the drugs that target these molecules, the discovery of chemokines suggested the development of small molecules and inhibitors with the desired specificity characteristics. Although selectin, integrin, or chemokine receptor blockade has proved highly effective in animal models of disease, the transfer of these results to human diseases has not yet been successful. Promising therapies have nonetheless been developed that target molecules involved in leukocyte trafficking. This is the case of anti-VLA-4 neutralizing antibodies (natalizumab) for multiple sclerosis therapy (111) and of anti-CCR9 compounds now in phase III clinical trials for treatment of Crohn’s disease and inflammatory bowel disease (IBD) (112, 113). The blockade of signaling pathways involved in leukocyte trafficking is also being explored, with promising results. JAK inhibitors are showing utility in clinic (114), mainly because they regulate cytokine-mediated leukocyte activation, although a potential effect on cell migration should also be considered (115). Other inhibitors that target antigen-mediated B and T cell activation (Syk inhibitors) also show positive results in phase III trials (116). Given their roles in cell proliferation and survival and in macrophage, B cell, mast cell and neutrophil activation, PI3K, and Bruton’s tyrosine kinase (BTK) inhibitors are also candidates for therapy (117, 118).

## Chemokines as Target of RA

Due to their central role in the selective recruitment and activation of immune cells at the inflammation site, chemokines and chemokine receptors are currently considered to potential therapeutic targets in several chronic autoimmune disorders. Inducible and homeostatic chemokines are heavily expressed in RA joints, produced mainly by activated synovial tissue and infiltrating leukocytes (119); elevated levels of several chemokines and their receptors are detected in serum and synovial fluid of RA patients (12) (Figure 2). Their relevance in disease progression has been determined in various animal models of the disease. Chemokines are implicated in RA development through recruitment and retention of different leukocyte populations in the inflamed joint (120, 121), but also elicit functions that contribute to pathogenesis. Chemokines can induce cytokine and metalloprotease release by chondrocytes and synovial fibroblasts, which contribute to cartilage destruction (122, 123). Other functions include induction of human chondrocyte death (124), enhanced cell proliferation (12, 125), angiogenesis, and angiostatic activities.

As indicated above, Th17 cells contribute to initiation and inflammatory phases of RA. Although Th17 cells express other chemokine receptors such as CCR4, CCR10, and CXCR3 (126, 127), they are characterized by CCR6 expression. CCL20, the CCR6 ligand, is a selective chemoattractant for T cells, naïve B cells, and immature DCs. CCR6^+^ Th17 cells have been identified in peripheral blood, synovial fluid, and inflamed tissue (128). CCL20 is expressed strongly at the inflamed joint, which allows Th17 cell activation and migration to the arthritic joint at early stages of the disease. Expression of other chemokine receptors in CCR6^+^ Th cells is associated with the expression of specific sets of cytokines. CCR4^+^/CCR6^+^ Th cells express high IL-17A levels, whereas levels of this interleukin are low in CXCR3^+^/CCR6^+^cells, whose IFN-γ levels are high. CCR6^+^/CCR10^+^ Th cells express high levels of IL-22, which defines the Th22 cell population. Other chemokine receptors found in CCR6^+^ Th cells are CCR5, CXCR4, and CXCR6, although they have not been associated with specific cytokine profiles. This cytokine production attracts and activates other cell types to the site of inflammation, including monocytes, neutrophils, synovial, and osteoclasts, which contribute to disease progression (128). Given this cytokine production, the induction of inflammatory chemokines during RA progression is not surprising.

In most cases, IL-1β- and TNF-α-activated cell types in the inflamed joint induce chemokine expression, although other cytokines such as IL-17 and IFNγ were also shown to upregulate expression of several chemokines. IL-1 and TNF-α stimulation of cells induce high CXCL8 levels in synovial tissue and fluid of inflamed joints (129, 130), and anti-CXCL8 treatment prevents neutrophil infiltration and tissue damage in LPS/IL-1-induced arthritis in mice (131). Production of CCL13, a major chemoattractant for eosinophils, T cells, and monocytes, is enhanced in cartilage by IFNγ, IL-1β, and TNF-α stimulation. As anticipated, the expression of these chemokines correlates with the recruitment of cells that express their receptors to the inflamed joint (132) (Figure 2).

CCL2 is also upregulated in synovial tissue of RA patients (130). It is produced by chondrocytes and synovial fibroblasts and can recruit CCR2^+^ macrophages to the synovia, as well as T cells, NK cells, and basophils (133, 134). Injection of a specific neutralizing anti-CCL2 mAb into rats with CIA reduced ankle swelling associated with decreased macrophage numbers in the joints (135); similar treatment inhibited arthritis in a MRL-lpr mouse model (136). Nonetheless, targeting CCL2 is not always valuable, and anti-CCL2 mAb treatment during the progression phase of a murine CIA model aggravated RA (137). Results were also discouraging in CIA models developed in mice that lacked the CCL2 receptor, CCR2 (138, 139). CCL3 and CCL5 are both expressed by activated T cells stimulated with IL-1β and TNF-α, by fibroblast-like synoviocytes, and by mononuclear cells in RA synovium (140–143); targeting their receptor, CCR5, could be of interest in pathological conditions. Whereas the percentages of CCR1^+^ and CCR5^+^ monocytes are lower in RA patient peripheral blood compared with normal controls, synovia of these patients show abundant CCR1 and CCR5 expression, indicating upregulation of these receptors and/or accumulation of CCR1^+^ and CCR5^+^ cells in the synovial compartment (144, 145). In mice, subcutaneous treatment with a CCR5 antagonist initiated a few days before clinical signs of arthritis promoted a marked decrease in leukocyte migration to joints, and thus reduced disease incidence and severity (146). Suppression of joint inflammation, reduced joint destruction, and diminished disease development was observed in CIA in rhesus monkeys treated with a CCR5 antagonist (147). These data are in agreement with reports showing that CCR5 density on the T cell surface determines the efficiency of T cell attraction to the joint, which might explain intra-individual variability and resistance of Δ32-CCR5 individuals to RA development (148, 149).

The CXC chemokine also have a role in RA due to their chemotactic effects on cell populations such as neutrophils (CXCL1, CXCL5, CXCL8), monocytes, and T cells (CXCL4, CXCL9, CXCL10, CXCL12, CXCL16), which correlates with the presence of CXCR3^+^ T cells, recruitment of CXCR6^+^ Th1 effector cells, and accumulation of CD4^+^ T cells in the RA synovium (143). There is growing evidence of an important functional role for CXCR4/CXCL12 in T lymphocyte accumulation and positioning within the rheumatoid synovium. CD4^+^ T cells in the inflamed synovium express high CXCR4 levels, which tallies with the high CXCL12 concentration in RA patient synovia (121) and suggests that CXCR4 is important for T cell retention in RA-affected synovial tissues (120). This is further supported by the observation that Th1 cells are attracted by RA synovial fluid, and that this chemotaxis can be inhibited *in vitro* by anti-CXCL12 antibodies (150). These studies show that CXCL12 production and CXCR4 expression are responsible for the characteristic pattern of T lymphocyte accumulation seen in the rheumatoid synovium (Figure 2). In accordance with the role of CXCL12/CXCR4 in RA, several CXCR4 antagonists, including the binding site competitor AMD3100, have shown therapeutic activity in arthritic mice (151).

In contrast to CC chemokine, the CXC group can also participate in angiogenic or angiostatic effects in RA patient joints. Synovia from RA patients show increased numbers of blood vessels compared to healthy synovium (12). It is generally thought that the new vessels accommodate the influx of immune cells into the joint and thus contribute indirectly to cell infiltration. RA synovium can show certain histological similarities to lymphoid tissue, including the presence of germinal centers, B cells, T cells, and follicular DCs. This could be due to the induction of newly expressed homeostatic chemokine receptors by the local microenvironment once T cells have entered the synovium, together with local expression of matching chemokines. Consistent with this hypothesis, CCR7 and CXCR4 are expressed by CD4^+^ memory T cells in RA synovial fluid, whereas circulating CD4^+^ T cells do not express these receptors (152, 153). Homeostatic chemokines that regulate cell traffic in lymphoid tissues are similarly found in the RA synovia, including CCL19, CXCL12, and CXCL13, and therefore can also participate in this lymphoid-like organization (154).

Although considered an initially promising therapy, results for blockade of chemokines or chemokine receptors in patients have been disappointing (155). A mAb against CCL2, the CCR2 ligand, showed no beneficial effects when administered to RA patients (156). Similarly, anti-CXCL8/IL-8 antibody treatment did not lead to clinical improvement in RA patients (157), and although short-term treatment of active RA patients with a CCR1 antagonist showed a tendency toward clinical improvement compared to controls (158), a phase II clinical study did not demonstrate clinical efficacy after a 6-month treatment (159).

Chemokine biology is more complex than originally anticipated. In addition to their considerable promiscuity and redundancy, the chemokine receptors oligomerize at the cell membrane (160, 161). This oligomerization is not limited to other chemokine receptors, as they can also interact with other GPCR (162, 163) and with other cell surface molecules such as CD4 (164). Chemokine signaling requires preformed receptor dimers (165) that allow G protein coupling to the receptor and activation of G protein-dependent and -independent signaling pathways. Chemokine receptor complexes help to generate diversity in chemokine signaling and function (160, 166, 167). In a complex microenvironment such as that of the inflamed arthritic joint, chemokine receptors are co-expressed, and chemokines and cytokines are upregulated. The lack of drugs that target chemokine receptors efficiently might also reflect greater complexity of the system than initially predicted and indicate that efficient chemokine inhibition could require additional therapeutic approaches that regulate interactions between chemokines, and between chemokines and cytokines that recruit proinflammatory cells to the arthritic joint. In addition, *in vivo* secreted chemokines bind to GAG, allowing formation of chemotactic gradients that direct leukocytes to inflammation sites. CXCL12 attached to sulfate proteoglycans has been observed on endothelial cells of the RA synovium, a process upregulated by inflammatory cytokines (168). These findings indicate that both chemokine upregulation and the GAG-dependent immobilization of these mediators on endothelial cells are potential targets for intervention.

## Conclusion

During the course of RA, T cells and other immune cells are recruited to the synovial tissue, where they produce large amounts of proinflammatory cytokines and interact with synovial fibroblasts and macrophages, all of which contribute to pathogenesis development. These include CD4^+^ and CD8^+^ cells, mostly with an activated phenotype. RA was classically considered a Th1-mediated disease, but evidence today indicates clear involvement of Th17, Th22, and Treg cells; it nonetheless remains unclear whether these are truly separate subpopulations or they represent plasticity and heterogeneity within the Th17 lineage. Each of these cell subsets acts at distinct stages in the course of the disease, to participate in the complex network of cell–cell interactions that governs RA initiation and progression, including release of inflammatory mediators, induction of cell proliferation, and angiogenesis. Cell migration into the synovium is controlled by the expression of selectins and their ligands, integrins, adhesion molecules, and chemokines and their receptors; all these molecules define the specific T cell subsets in the inflamed joint. The use of antagonists to and mice deficient in these proteins has been essential for defining their role in different steps of the disease, and prompted the use of inhibitors in clinical studies. The diversity of chemokines and receptors suggested they were ideal targets that only affect specific leukocyte subsets, and over the last two decades, most pharmaceutical and biotechnology companies developed chemokine receptor-targeting reagents that were analyzed for RA therapy. These clinical studies were not as successful as anticipated and dashed the promise of targeting individual chemokine receptors for RA. Alternative strategies aimed at intracellular signaling pathways or interactions between chemokine receptors must thus be considered.

## Conflict of Interest Statement

The authors declare that the research was conducted in the absence of any commercial or financial relationships that could be construed as a potential conflict of interest.
